# Multimodal dataset on glucose interpretation, treatment decisions and smartwatch visualisation for type 1 diabetes

**DOI:** 10.1038/s41597-026-07189-0

**Published:** 2026-04-13

**Authors:** Rujiravee Kongdee, Bijan Parsia, Hood Thabit, Simon Harper

**Affiliations:** 1 Department of Computer Science, University of Manchester, Manchester, M13 9PL United Kingdom; 2 Department of Software Engineering, Nakhon Pathom Rajabhat University, Nakhon Pathom, Thailand; 3 Manchester Royal Infirmary, Manchester, M13 9WL United Kingdom

## Abstract

People with Type 1 Diabetes Mellitus (T1DM) must frequently check their blood glucose and make over 180 health-critical decisions daily alongside everyday life decisions. These individuals heavily rely on glucose monitoring devices to monitor their glucose levels, yet the user interfaces do not support accurate interpretation and treatment decision-making. The constant need to check glucose levels several times a day also contributes to negative emotional impacts, including diabetes distress and burnout, where individuals eventually disengage from self-care. This dataset comprises three complementary components: (1) transcripts capturing how 27 individuals with T1DM interpret and make treatment decisions presented on their familiar monitoring interfaces, (2) questionnaire responses from 86 individuals with T1DM detailing their treatment actions across various glucose scenarios and (3) 11 smartwatch interfaces designed to support ambient, at-a-glance glucose awareness. Together, this dataset provides an in-depth investigation of interpretation errors, decision-making processes, and design opportunities. The dataset can support clinicians in understanding patient reasoning and provides researchers with a foundation for developing glucose interfaces that support better long-term diabetes self-management.

## Introduction

### Background

T1DM is a chronic, incurable condition caused by the destruction of pancreatic beta cells responsible for insulin production, leading to the body’s inability to produce insulin^1,2^. Without insulin, glucose accumulates in the bloodstream, requiring lifelong insulin therapy. Clinically recommended blood glucose levels range between 3.9 and 10.0 mmol/L, referred to as in-range glucose^3,4^. Levels above 10.0 mmol/L indicate hyperglycaemia, while levels below 3.9 mmol/L indicate hypoglycaemia^1,5^.

Managing T1DM is highly challenging due to unpredictable glucose fluctuations. Individuals make an estimated 180 additional daily decisions to manage their condition^6^, influenced by at least 42 known factors such as diet, activity, medication, behaviour, environment, and biological variability^7^. For example, meal planning requires evaluating food type, quantity, timing, and insulin dosage^8,9^. These constant cognitive and behavioural demands are critical, as errors can lead to hypo- or hyperglycaemia, highlighting the complexity and importance of daily T1DM management.

Currently, three primary technologies are used to monitor glucose levels in people with T1DM: Real-time Continuous Glucose Monitoring (CGM), Intermittently scanned CGM (Flash), and Self-Monitoring of Blood Glucose (SMBG)^10,11^. Individuals with T1DM rely on these devices to frequently check their glucose levels and make safety-critical treatment decisions to maintain glucose within a safe range. CGM and Flash devices are widely adopted because they continuously measure glucose and present real-time data through mobile applications. However, prior research has reported usability issues with these interfaces. Specifically, they have been shown to demand high cognitive effort, be difficult to interpret, and require considerable time to extract meaningful information^12–14^. These limitations are particularly concerning, as misinterpretation or delayed interpretation of glucose data can lead to inappropriate treatment decisions and, in extreme cases, life-threatening outcomes.

### Interpreting CGM data

Several valuable datasets capturing glucose data from people living with T1DM have been made available in recent years^15–18^. These datasets collect a wide range of information, including data from glucose monitoring devices. While they are highly useful for developing advanced algorithms to support glucose management, there remains a lack of datasets that capture how people with T1DM interpret data from glucose monitoring device user interfaces and translate it into treatment decisions during everyday use. Therefore, we would like to share the data we collected from our studies, which involved an in-depth investigation with 27 people with T1DM to understand how they interpret 8 - 10 glucose scenarios presented on their everyday monitoring interfaces. Furthermore, we also gathered treatment decisions that 86 participants made on 12 - 14 glucose scenarios.

We consider this dataset worth sharing, as it reveals several insights that the interfaces might be less interpretable than expected. For example, we found that the accuracy in correctly interpreting the interfaces is less than 40%, and less than 22% in making treatment decisions. Moreover, we observed responses indicated they would seek medical or emergency support in several non-critical glucose scenarios. Despite this, 75% of them reported high confidence in self-management, and 85% reported that they strongly followed clinical guidelines^19^. This discrepancy between actual performance and perception is particularly concerning, as it suggests that inappropriate treatment decisions may occur unknowingly and persist over extended periods, increasing the risk of adverse health outcomes.

In response to these findings, we have made the collected data publicly available to maximise its utility and impact. This dataset provides transcripts of how people with T1DM interpret glucose data and make treatment decisions based on real-world monitoring interfaces, insights that are difficult to obtain during clinical visits. For clinicians, the dataset can support a deeper understanding of where and how these individuals misinterpret glucose information, and the extent to which their decision-making deviates from clinical recommendations. These insights can inform more targeted clinical conversations and personalised education strategies. Beyond clinical practice, this dataset can be used to inform the development of improved training and educational materials, particularly for newly diagnosed individuals. By highlighting common misinterpretations and decision-making errors, the data can support training that is grounded in real user behaviour rather than assumed understanding.

From a human-computer interaction perspective, this dataset offers a valuable empirical foundation for future research into glucose data visualisation and interface design. It can help researchers to explore alternative visualisation approaches that are specifically tailored to the T1DM context, rather than using standard data visualisations that may not adequately support interpretation or decision-making^12,13^. As such, the dataset can facilitate the design and evaluation of more interpretable and decision-supportive interfaces.

In addition, we provide mock-ups of all glucose scenarios used in our studies. The mock-ups could be used in several ways. First, it could be used to replicate our study to examine how people with T1DM interpret them under different settings, such as a larger sample size, different participants’ demographics or different glucose management. Second, future work could use these mock-ups to investigate how people with T1DM make sense of them using different research methods than those we used. Finally, the mock-ups can serve as practical educational resources for T1DM training, supporting scenario-based learning to help individuals practise interpreting glucose data and making appropriate treatment decisions.

### Emotional burden from constant monitoring

On the emotions side, research shows that constant glucose monitoring is often burdensome, with many individuals reporting heightened anxiety and stress tied to this relentless responsibility. Out-of-range glucose values are not only numbers on a screen but can produce deep feelings of frustration, discouragement, and even personal failure^13,20^. Some people with T1DM have described a visceral reaction, such as nausea, simply from viewing an out-of-range glucose graph, even in the absence of physical symptoms^21^. Over time, this persistent emotional strain and diabetes management distress can dramatically lead to diabetes burnout and, in some cases, disengagement from self-management altogether^22–24^.

Building on these emotional challenges, we collaboratively developed a series of smartwatch face prototypes with people living with T1DM using the principle of glanceable visualisation. This approach introduces a new paradigm for presenting glucose information in the T1DM context, in which data are delivered ambiently and subtly, which allows users to remain informed without being overwhelmed. This glanceable visualisation has the potential to mitigate the emotional burden associated with continuous monitoring while still supporting timely awareness of glucose trends. All prototypes developed during the co-design sessions are included as part of this dataset to support reuse and further exploration of this design space. These prototypes can be used as practical guidelines for designing new smartwatch faces or as a foundation for developing alternative glanceable interfaces.

This dataset also provides the source code for the evaluation of glanceable visualisations, which other researchers can use to assess the glanceability of smartwatch faces beyond the prototypes presented in this study. Although the evaluation was conducted using a simulated programme, it can support feasibility testing and controlled usability evaluations, particularly in situations where access to physical devices or the ability to conduct field studies is limited.

Taken together, this dataset provides resources on how people with T1DM interpret glucose information and make treatment decisions when using glucose monitoring device interfaces, alongside data supporting a new design paradigm based on glanceable visualisation to alleviate the emotional burden of continuous monitoring. The data were collected across a series of interconnected studies and provide a valuable resource for research in the context of T1DM management and human-computer interaction.

## Methods

We organised the data into three main parts: (1) T1DM Interpretation, (2) T1DM Treatment Decision-making, and (3) T1DM Glanceable Visualisation. Each part corresponds to one of the conducted studies. All methods from these three parts were carried out in accordance with the institutional guidelines and regulations. This section outlines how data from each part was collected.

### Part 1: T1DM Interpretation

In this study, we aimed to gain an in-depth understanding of (1) how people with T1DM interpreted their daily use of glucose monitoring device user interfaces and (2) what treatment decisions they made based on these interfaces. To capture their immediate responses, we used a Think Aloud protocol^25^ as our semi-structured interview method. Participants were presented with one glucose monitoring user interface at a time, and we used Microsoft PowerPoint to display the interface. Following each interface, they were asked to verbally express their interpretations and indicate the treatment action they would take if the presented interface were their own. This was elicited through the questions: “Can you tell me what is going on on this screen?” and “What would you do if this screen were yours?”.

Responses from participants were evaluated against standard clinical guidance, the National Institute for Health and Care Excellence (NICE)^26^. The level of accuracy of each response was based on the alignment with the guidance, which was categorised into 4 levels: (1) All correct, (2) Minor mistake, (3) All incorrect, and (4) Not mentioned. To analyse the overall accuracy, we used a weighted average where we gave the weights of 2, 1, 0, and 0 to each level, respectively.

Prior to the interview session, they received a brief training session and were allowed to respond without any time constraints. The interview sessions were conducted online via the Zoom platform. To comply with institutional ethical regulations, no audio or video recordings were made. Instead, participants’ speech was transcribed in real time into text using the tool *tl;dv*.

The user interfaces presented to participants were developed to mimic the user interfaces of the most-used glucose monitoring devices and are prescribed by the National Health Service (NHS) in the UK: Dexcom G6 (CGM), FreeStyle Libre 2 (Flash), and Contour Next One (SMBG)^27,28^. As we did not have access to real patient glucose data, we designed the user interfaces to represent a wide range of glucose scenarios. The scenarios covered: level 1 hypoglycaemia (3.4-3.8 mmol/L), level 1 hyperglycaemia (10.1-13.9 mmol/L), in-range glucose (3.9-10.0 mmol/L), significantly high glucose (>22.2 mmol/L), significantly low glucose (<2.8 mmol/L), high glucose (14.0-22.2 mmol/L), low glucose (2.8-3.3 mmol/L), and glucose low alerts (2.9 mmol/L within 20 minutes).

After the interview, a survey consisting of ten questions was administered to gather participants’ opinions regarding their self-management practices. The full list of survey questions is presented in Table 3. The survey was developed and distributed via the Qualtrics platform. Responses were collected using a 5-point Likert scale.

### Part 2: T1DM Treatment Decision-making

This study was a subsequent study of Part 1: T1DM Interpretation. In this study, we presented 86 participants with 12 - 14 glucose monitoring user interfaces, and they were asked to specify their treatment decisions, in which we asked, “What would you do if this screen were yours?” This study was conducted as a survey via the Qualtrics platform in which participants provided free-text responses with unrestricted length. We configured the survey to display one user interface at a time with random order. Following each interface, a text box was positioned below, allowing participants to type their responses.

The user interfaces presented to participants were developed to mimic Dexcom G6 (CGM) and FreeStyle Libre 2 (Flash) interfaces. Participants were shown a user interface representative of their current device. In terms of glucose scenarios, we aimed to cover more scenarios than in the previous study, which included: level 1 hypoglycaemia (3.4-3.8 mmol/L), level 1 hyperglycaemia (10.1-13.9 mmol/L), in-range glucose (3.9-10.0 mmol/L), significantly high glucose (>22.2 mmol/L), significantly low glucose (<2.8 mmol/L), high glucose (14.0-22.2 mmol/L), low glucose (2.8-3.3 mmol/L), urgent low soon alert (3.1 mmol/L within 20 minutes), urgent low alarm (<3.9 mmol/L), and sensor error. Each category was represented with two interfaces.

Please note that this study initially aimed to compare the correctness of participants’ choices of treatment decision between the control group and the experimental group. The control group was presented with mimicked CGM and Flash interfaces, while the experimental group was presented with modified user interfaces incorporating a textual meaning of the components displayed on the interfaces: glucose graph, glucose number, trend arrow and alert banner. More detailed information is reported in a separate work^29^.

### Part 3: T1DM Glanceable Visualisation

In this study, we collaborated with four individuals with lived experience of T1DM to co-design smartwatch face prototypes grounded in the principles of glanceable visualisation^30^. Together, we developed ten prototypes.

We applied two established co-design frameworks^31,32^ across five phases, with the final phase (evaluation) conducted separately in another study. The phases were as follows:

1. **Pre-design:** defined the objectives of the sessions, focusing on creating glanceable glucose visualisations for smartwatch faces and understanding how people with T1DM currently engage with their data.
2. **Generative:** co-designers identified key glucose components for display and created low-fidelity prototypes.
3. **Prototyping:** the researcher (RK) converted low-fidelity concepts into high-fidelity prototypes.
4. **Evaluative:** co-designers reviewed the high-fidelity prototypes, provided feedback, and suggested modifications, which the researcher refined.
5. **Post-design:** final prototypes were evaluated. The experimental study of this phase was carried out and reported in a separate study^33^.

A total of ten prototypes were developed. Co-designers subsequently voted for their five most preferred prototypes, from which two emerged as the most frequently selected. These two prototypes were subsequently evaluated in our pilot study. In addition to these two prototypes, the research team independently developed a third prototype to serve as a comparator.

Regarding the evaluation, a web-based simulated system was developed to assess the glanceability of each prototype. This system simulated real-world activity where participants were watching a video on their laptop while wearing a smartwatch that they could glance at to see their glucose status. We captured data on participants’ interactions, specifically noting when they viewed the smartwatch face and the duration of each view.

### Ethical approval

Each dataset corresponds to a separate study, and the ethical approvals for each are outlined below:

1. **T1DM Interpretation**^19^: Participants were recruited from online diabetes communities and through physical poster distribution on the University of Manchester campus. To address limitations associated with in-person consent due to remote participation, verbal consent was obtained via the Zoom platform (version 5.16.0). Participants reviewed the consent form displayed on the screen, and their verbal agreement was documented. The researcher ticked the consent box on their behalf after receiving explicit permission. All participants provided written informed consent to participate in this study, including permission for publication and the open sharing of their anonymised data. This study received an official ethical exemption approval from the Research Governance, Ethics and Integrity Department and the University Research Ethics Committees at the University of Manchester (Reference number: 2023-17960-30585). No identifiable data were collected in either the qualitative interviews or the survey studies.
2. **T1DM Treatment Decision-making**^29^: Participants were recruited by posting an advertisement in online diabetes communities and various advertising platforms provided by the JDRF, the Type 1 Diabetes Research Charity, UK. All participants provided written informed consent to participate in this study, including permission for publication and the open sharing of their anonymised data. This study received an official ethical exemption approval from the Research Governance, Ethics and Integrity Department and the University Research Ethics Committees at the University of Manchester (Reference number: 2024-20659-35490). No Participant Identifiable Information data was collected during this study.
3. **T1DM Glanceable Visualisation**^30,33^: The co-design part of the study was classified as Patient and Public Involvement and Engagement (PPIE) activity, as it involved collaborative work with members of The T1DM Brains Trust, University of Manchester. As such, it was ethically exempted by the Research Governance, Ethics and Integrity Department and the University Research Ethics Committees at the University of Manchester (Reference number: 2025-23706-41879). All participants provided written informed consent to participate in this study, including permission for publication and the open sharing of their anonymised data.

## Data Records

The dataset is available in a Zenodo Repository ‘GIMA-T1DM-Dataset’ at 10.5281/zenodo.17342854^34^.

### Dataset structure

**Fig. 1:**
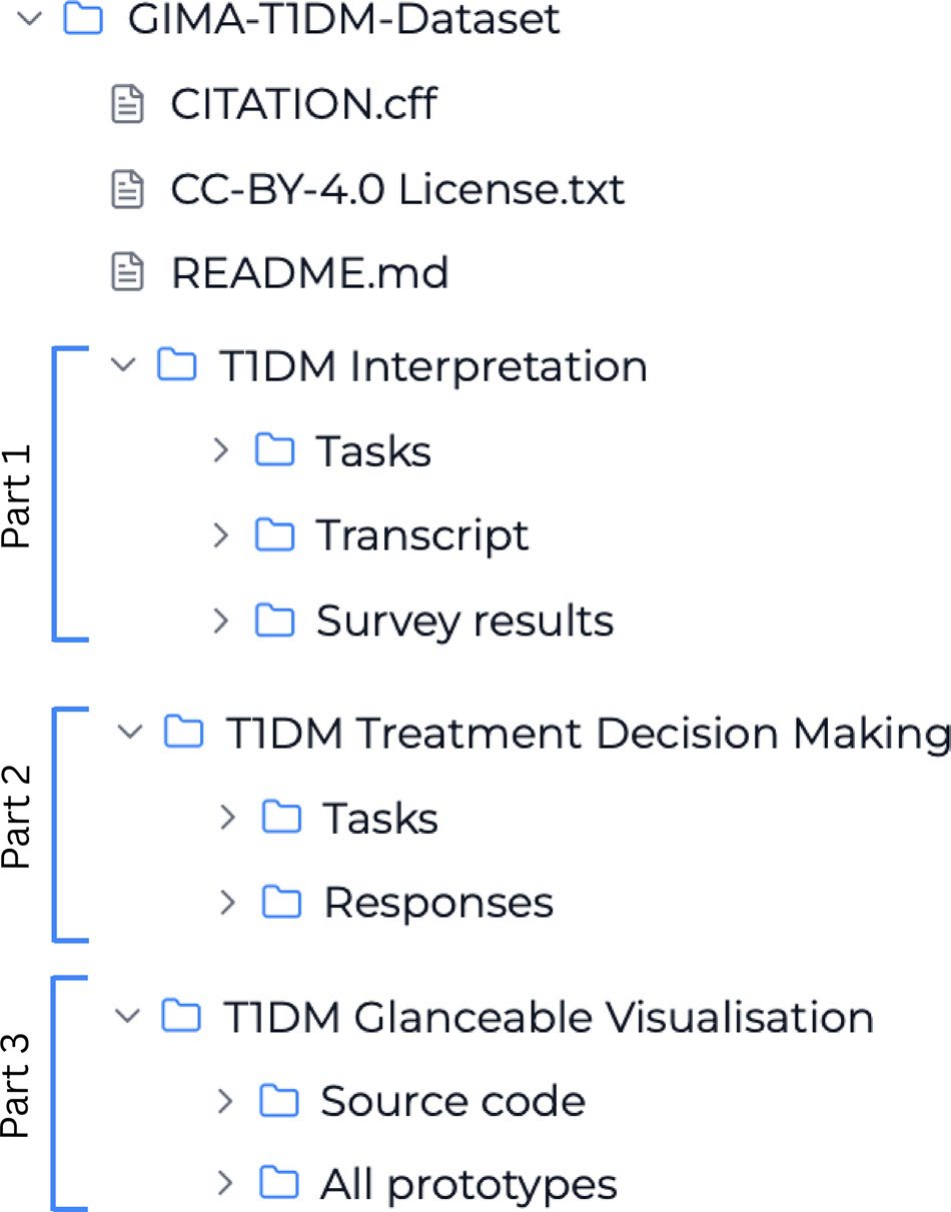
Dataset folder structure containing three metadata files and three subfolders: T1DM Interpretation, T1DM Treatment Decision Making and T1DM Glanceable Visualisation.

The complete dataset folder structure is illustrated in Fig. 1. The root folder, **GIMA-T1DM-Dataset**, contains three metadata files and three main subfolders, detailed as follows:

- **CITATION.cff**: a plain-text metadata file to specify how this work should be cited.
- **CC-BY-4.0 License.txt**: contains the Creative Commons Attribution 4.0 International License. This file specifies that the contents of the repository may be used, shared, and adapted for any purpose, including commercial use, provided proper credit is given to the original author.
- **README.md**: a markdown file providing an overview of the project, instructions for use, and other relevant details such as setup, features, and licensing.
- **T1DM Interpretation**: contains all mock-ups used in the study, participant transcripts, and survey results.
- **T1DM Treatment Decision Making**: contains all mock-ups used in the study along with participants’ survey results.
- **T1DM Glanceable Visualisation**: contains all prototypes developed in the study, as well as the source code for the simulated evaluation program.

### Part 1: T1DM Interpretation

This section presents collected data from our first study in the series, investigating glucose monitoring user interface misinterpretation in people with T1DM^19^. Participants’ demographic is also presented.

#### Participant information

**Fig. 2:**
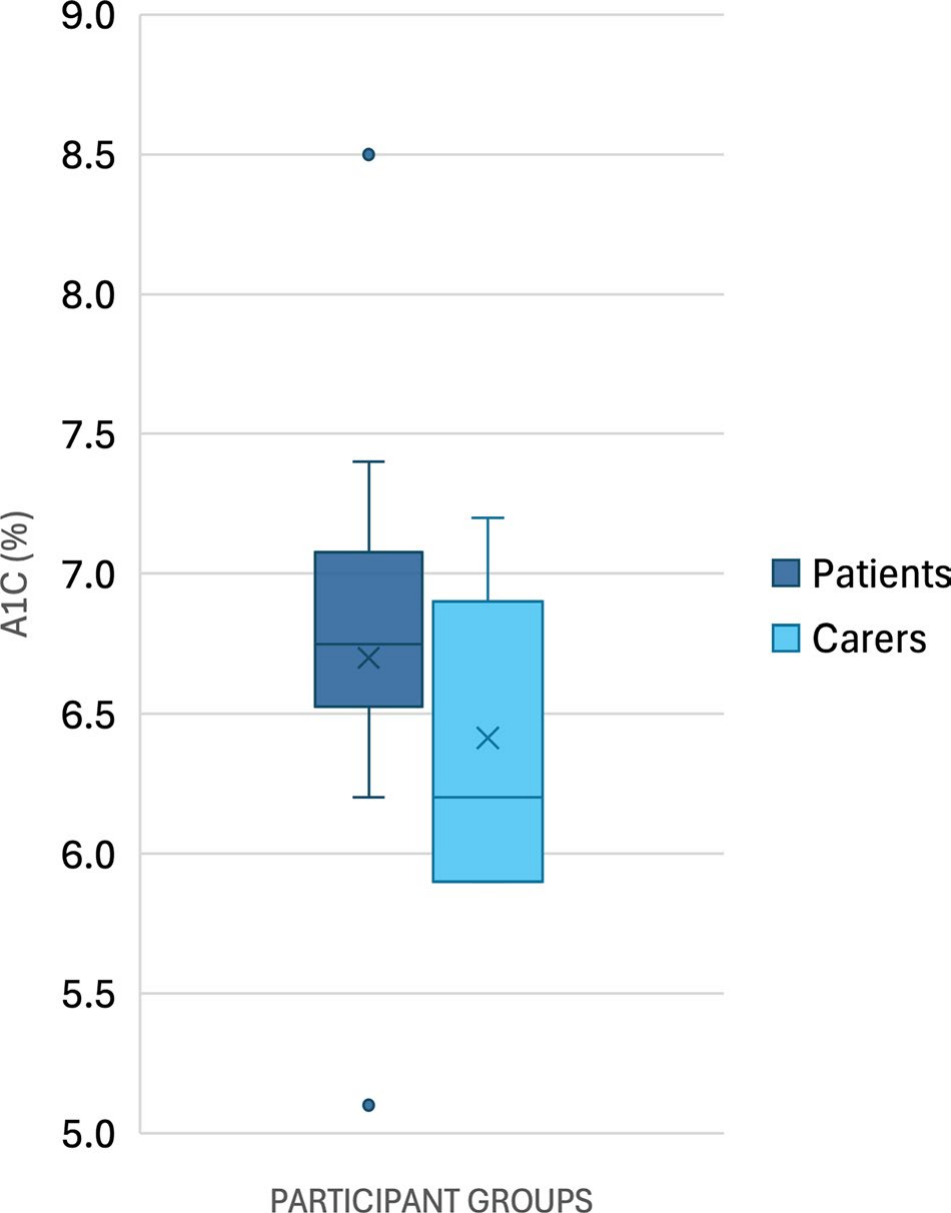
A1C distribution of participants in Part 1: T1DM Interpretation.

Table 1 summarises participant demographics. A total of 27 participants took part in the study, comprising 17 patients and 10 carers. Figure 2 depicts A1C among study participants. Eligibility criteria were as follows: clinically diagnosed with T1DM for more than two years or providing care for a person diagnosed with T1DM; aged ≥18 years; use of a CGM, Flash, or SMBG device; and the ability to speak and read in English. Participants were recruited through online diabetes communities and via physical poster distribution on the University of Manchester campus.

**Table 1:** Participant demographic information for Part 1: T1DM Interpretation.

	Patients (*n* = 17)	Carers (*n* = 10)
**Age**		
18–24	0	4
25–39	16	6
40–60	1	0
**Gender**		
Male	16	7
Female	1	3
**Device**		
Dexcom G6	3	6
FreeStyle Libre	8	1
Glucometer	6	3
**Insulin therapy**		
Insulin pen	2	1
Insulin pump	13	9
Both insulin pen and pump	2	0
**Duration of diabetes**		
2–5 years	3	2
6–10 years	5	4
> 10 years	9	4
**Familiarity with graph/dashboard**		
Not at all	0	0
Slightly familiar	5	2
Moderately familiar	5	4
Very familiar	6	4
Extremely familiar	1	0
**A1C**		
%	6.7 ± 0.8	6.4 ± 0.5
mmol/mol	49 ± 8	46 ± 5

#### Description

This folder contains three subfolders: (1) **Tasks**, (2) **Transcripts**, (3) **Survey results** and (4) **Word counts**. Table 2 summarises the contents of these subfolders.

**Table 2:** T1DM Interpretation folder descriptions.

Folder name	Size	Number of files	Description
Tasks	2.8 MB	10	Contains 10 mock-ups representing each task
Transcripts	553 KB	27	Contains 27 transcripts from 27 participants
Word counts	12 KB	1	Contains word counts of each participant’s transcript
Survey results	16 KB	1	Contains the survey results in 1 file

**Fig. 3:**
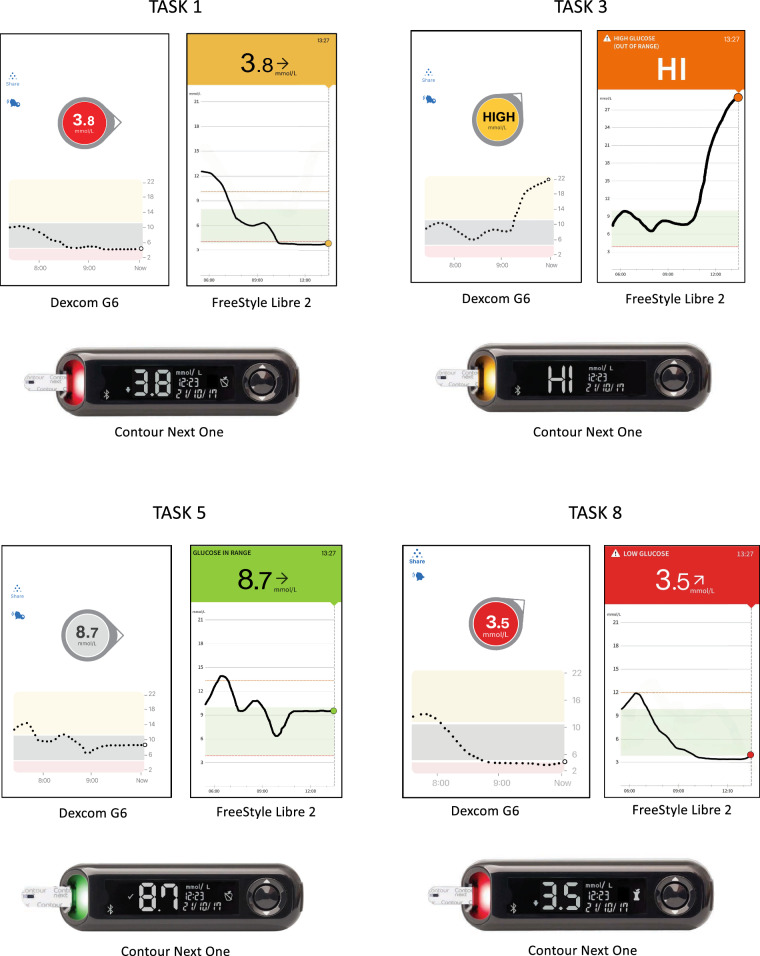
Example mock-ups representing different glucose scenarios from Part 1: T1DM Interpretation.

The **Tasks** folder contains all CGM, Flash, and SMBG mock-ups manually developed to represent different glucose scenarios. There are 10 tasks for CGM and Flash and 8 tasks for SMBG. All images are provided in PNG format. An example of the task images is shown in Fig. 3. The **Transcripts** folder contains participant transcripts derived from the semi-structured interview sessions, stored as DOCX files. Next, the **Word counts** folder contains one Excel spreadsheet file specifying word counts of each participant’s responses. Finally, the **Survey results** folder contains a single file, *Survey results*, which records participants’ opinions on their self-management practices. This file is provided as an Excel spreadsheet, with its description outlined in Table 3.

**Table 3:** Survey responses file description from Part 1: T1DM Interpretation.

Survey question	Data type
Q1: Did you have any diabetes education courses when you or your patient were diagnosed? (If yes, please specify the course names if you can remember.)	Text
Q2: What is the blood glucose range that you feel comfortable with?	Text
Q3: How many times do you or your patient have hypoglycaemia episodes per week?	Text
Q4: How worried are you or your patient about having hypoglycaemia?	7-point Likert
Q5: How worried are you or your patient about having hyperglycaemia?	7-point Likert
Q6: Do you know how your body or your patient’s body reacts to diabetes better than a clinician?	7-point Likert
Q7: How much do you or your patient follow the clinical guidance?	7-point Likert
Q8: How difficult is it for you or your patient to follow the clinical guidance?	7-point Likert
Q9: How anxious are you about your own or your patient’s diabetes management?	7-point Likert
Q10: How confident are you in your own or your patient’s diabetes self-management?	7-point Likert

### Part 2: T1DM Treatment Decision-making

This section presents the data collected in the second study, which captured participants’ responses to treatment decision-making across various glucose scenarios^29^. All developed user interfaces are also included.

#### Participant information

**Fig. 4:**
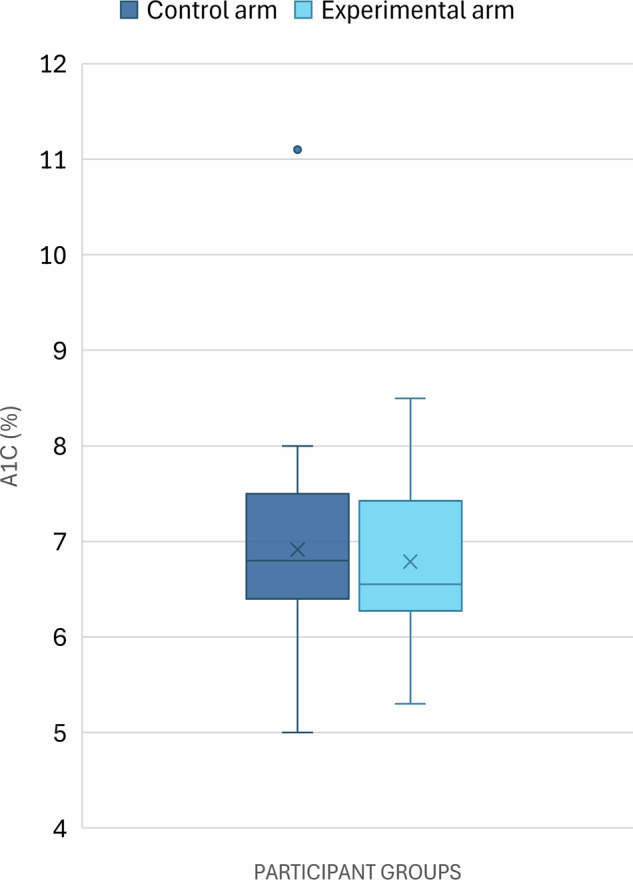
A1C distribution of participants in Part 2: T1DM Treatment Decision-making.

Table 4 summarises participant demographics. A total of 86 participants were recruited through advertisements posted in online diabetes communities and via advertising platforms provided by JDRF, the Type 1 Diabetes Research Charity, UK. Figure 4 depicts A1C among study participants. The inclusion criteria were: aged over 18 years; diagnosed with T1DM or being a carer of a person with T1DM; current use of either a Dexcom or a FreeStyle Libre device; and the ability to read and write in English.

**Table 4:** Participant demographic information for Part 2: T1DM Treatment Decision-making.

Demographic	Control arm (*n* = 44)	Experimental arm (*n* = 42)
**Device group**		
CGM users	16	14
Flash users	28	28
**Insulin regimen**		
Insulin pen/pump	42	41
Others (hybrid closed loop)	2	1
**Role**		
Patient	42	40
Carer	2	2
**Age group**		
18–30	8	6
31–60	31	30
≥ 60	5	6
**T1DM structured education experience**		
Yes	31	27
No	13	13
Not sure	0	2
**A1C (%)**		
Average	6.9 ± 0.9	6.8 ± 0.8

#### Description

This folder contains two subfolders: (1) **Tasks** and (2) **Responses**. Table 5 summarises the contents of these subfolders.

**Table 5:** T1DM Treatment Decision-making folder description.

Folder name	Size	Number of files	Description
Tasks	14.3 MB	2 sub-folders	Contains all mock-ups used in the study, separated into 2 sub-folders: Baseline and Meaning
Responses	74 KB	1	Contains 86 responses from 86 participants

**Fig. 5:**
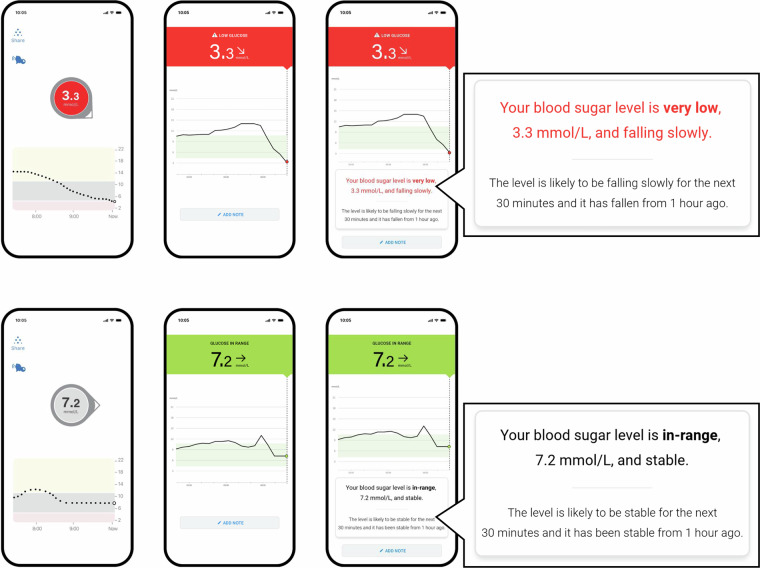
Example mock-ups representing different glucose scenarios from Part 2: T1DM Treatment Decision-making.

The **Tasks** folder contains all mock-ups representing different glucose scenarios for CGM and Flash interfaces used in the study. These are organised into *Baseline* and *Meaning* subfolders. The former contains the mock-ups presented to the control group, while the latter contains the mock-ups presented to the experimental group, as described above. Within each of these, further separated by CGM and Flash devices. There are 14 tasks for CGM and 12 tasks for Flash. All mock-ups are provided in PNG format. Examples are shown in Fig. 5.

The **Responses** folder contains a single file, *Participant responses*, which includes participants’ free-text responses to each glucose scenario presented, along with their preference and opinion towards the modified user interfaces. This file is provided as an Excel spreadsheet, with its description outlined in Table 6.

**Table 6:** *Participant responses* file description.

Column	Description	Data type
Device	Specify participant’s device: CGM or Flash	Text
Arm	Specify participant’s group: Baseline for Control group, Meaning for Experimental group	Text
Level 1 hypo	Participant’s responses to this glucose scenario	Text
Level 1 hyper	Participant’s responses to this glucose scenario	Text
Very low	Participant’s responses to this glucose scenario	Text
Very high	Participant’s responses to this glucose scenario	Text
HI	Participant’s responses to this glucose scenario	Text
LO	Participant’s responses to this glucose scenario	Text
Alert 1	Participant’s responses to this glucose scenario	Text
Alert 2	Participant’s responses to this glucose scenario	Text
Alarm 1	Participant’s responses to this glucose scenario	Text
Alarm 2	Participant’s responses to this glucose scenario	Text
Sensor error 1	Participant’s responses to this glucose scenario	Text
Sensor error 2	Participant’s responses to this glucose scenario	Text
In range 1	Participant’s responses to this glucose scenario	Text
In range 2	Participant’s responses to this glucose scenario	Text
Word counts	Number of word counts of each participant’s response	Number
Agree to text shown	Level of agreement to the add text	7-point Likert

### Part 3: T1DM Glanceable Visualisation

This section presents the dataset from our last study in the series, where we developed smartwatch prototypes based on the principle of glanceable visualisation^30^.

#### Participant information

In the co-design study^30^, four individuals living with T1DM were recruited, as summarised in Table 7. Recruitment was carried out via the T1DM Brains Trust email list at the University of Manchester and through posters distributed at Manchester Royal Infirmary. Eligibility criteria were: aged over 18 years; diagnosed with T1DM for more than two years; ability to participate using a laptop; and ability to communicate in English.

**Table 7:** Co-designer demographics from Part 3: T1DM Glanceable Visualisation.

	Age	Gender	T1DM duration	Glucose monitoring device	Smartwatch in use
Co-designer 1	18–30	Male	8 years	Dexcom CGM	Not using a smartwatch
Co-designer 2	≥60	Female	43 years	Medtronic CGM	Samsung Galaxy 23
Co-designer 3	31–50	Male	39.5 years	FreeStyle Libre Flash	Garmin (Fenix 6)
Co-designer 4	18–30	Male	10 years	Dexcom CGM	Apple Watch Ultra

#### Description

This folder contains two subfolders: (1) **All prototypes**, and (2) **Source code**. Table 8 summarises the contents of these subfolders.

**Table 8:** T1DM Glanceable Visualisation folder description.

Folder name	Size	Number of files	Description
All prototypes	3.5 MB	11 sub-folders	Contains all prototypes developed in the study (1 prototype per folder)
Source code	16.2 MB	1 sub-folder, 4 files	Contains source code of the simulation program

The **All prototypes** folder contains ten co-designed smartwatch face prototypes and one researcher-developed prototype, all in PNG format. The **Source code** folder includes the code for a web-based simulated system designed to evaluate the glanceability of each prototype. This system replicates a real-world scenario in which participants engage in an activity while wearing a smartwatch and raise their wrist to view glucose levels displayed on the watch face.

**Fig. 6:**
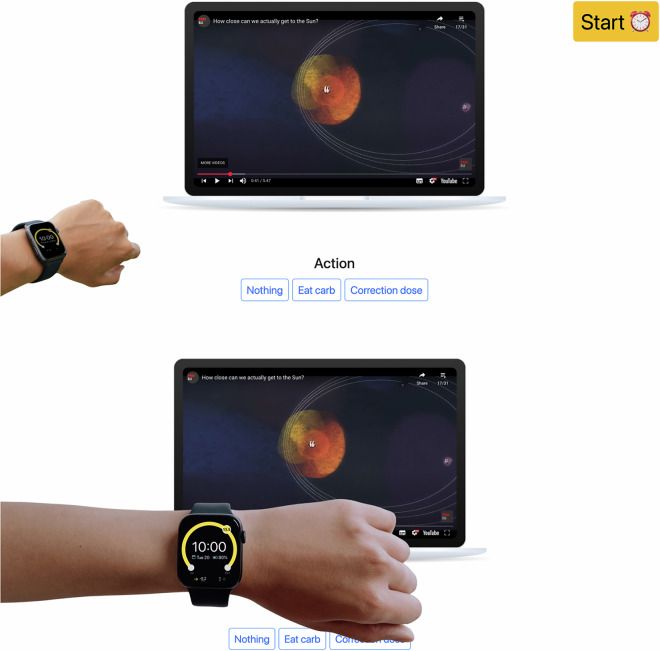
Screenshot of the system’s user interface we developed for this study. (Top) shows the simulated hand at rest. (Bottom) shows the hand when a participant views a watchface.

Regarding the **Source code** folder, it contains a website-based system we developed to evaluate the glanceability of each prototype. Figure 6 presents the system’s user interface. At the centre, a laptop screen displays a series of YouTube videos. In the bottom left corner, a hand wearing a smartwatch is shown to simulate a person’s hand resting beside the laptop, enabling participants to view the watchface at a glance. At the bottom of the system interface, three buttons represent treatment options that participants can select in response to the glucose level displayed at that moment. Participants can press the ‘A’ key on their keyboard to raise the simulated hand and view the smartwatch face. The system logs these interactions at one-second intervals. When a participant presses ‘A’, the system records this event as 1, along with the corresponding timestamp of the second in which the interaction occurs.

The system’s algorithm was set up with a predefined timeline of glucose scenarios spanning 1.5 hours, with glucose levels updated at 5-minute intervals. Each 5-minute interval corresponded to 60 seconds of real-time, resulting in an 18-minute study duration. As a result, the interaction data contains 1,080 data points. Please refer to our study^33^ for results of the experiment.

## Technical Validation

### Participants’ transcripts

In the study in Part 1, we conducted semi-structured interviews with participants and used a tool *tl;dv* to transcribe their verbal responses into textual responses. After each interview session, we immediately reviewed the generated transcript by reading all the generated text to see whether there were any missing data or illegible text. Moreover, we also needed to confirm that no personally identifiable participant data were recorded. Throughout this process, we merely reviewed the transcript without removing or altering any content. Following this, no missing data were observed, and the observed errors in the transcripts were syntactic and did not affect the analysis. Therefore, we did not make further adjustments.

Regarding the participants’ response evaluation, we ensured the correctness and consistency by performing the scoring process with the research team and further consulting with a clinical diabetologist from the Manchester Royal Infirmary to confirm the accuracy of the evaluation.

Although the Think Aloud method provided spontaneous responses from participants, it might introduce unnatural behaviour as participants were required to verbalise their thoughts continuously while reading the interface. In addition, participants might have responded that appeared appropriate but did not fully reflect how they would interpret or use the interface in their everyday lives.

### Survey responses

Regarding survey responses we collected in Part 1 and Part 2 studies, all surveys were distributed via the Qualtrics platform. We downloaded the responses directly from the platform in the form of a Comma-Separated Values (CSV) file. However, the platform automatically generated metadata columns such as record dates and response IDs in the responses file, which we removed as they were irrelevant. Throughout this process, we did not change or modify any responses. In addition, Likert scale responses were converted from numerical values to their corresponding labels. For example, *7* was converted to *Strongly agree* and *1* to* Strongly disagree*.

The use of a survey reached a large group of participants. However, one limitation remained. Since we accepted free-text responses from participants, it could lead participants to provide answers that may not accurately reflect the decisions they would make in real-world situations.

## Data Availability

The dataset is available in a Zenodo Repository ‘GIMA-T1DM-Dataset’ at 10.5281/zenodo.17342854^34^.

## Code availability

The source code in the T1DM Glanceable Visualisation part is available in a Zenodo Repository ‘GIMA-T1DM-Dataset’ at 10.5281/zenodo.17342854^34^.
